# Disproportionate Vertebral Bodies and Their Impact on Lumbar Disc Herniation

**DOI:** 10.3390/jcm10143174

**Published:** 2021-07-19

**Authors:** Ralph Läubli, Robin Brugger, Tatiana Pirvu, Sven Hoppe, Dominik Sieroń, Karol Szyluk, Christoph E. Albers, Andreas Christe

**Affiliations:** 1 Orthopedic Surgery, Interlaken Hospital FMI, Weissenaustrasse 27, 3800 Unterseen, Switzerland; rlaeubli@hotmail.com (R.L.); robin.brugger@insel.ch (R.B.); tatiana.pirvu@spitalfmi.ch (T.P.); 2 Wirbelsäulenmedizin Bern, Hirslanden Salem-Spital, Schänzlistrasse 39, 3000 Bern, Switzerland; sven.hoppe@hin.ch; 3 Orthopedic Surgery, Inselspital, Bern University Hospital, University of Bern, Freiburgstrasse 10, 3010 Bern, Switzerland; christoph.albers@insel.ch; 4 Silesian Center for Heart Diseases, Division of Magnetic Resonance Imaging, 41-800 Zabrze, Poland; 5 I Departament of Trauma and Orthopaedics, District Hospital of Orthopaedics and Trauma Surgery, Bytomska 62 str, 41-940 Piekary Śląskie, Poland; szyluk@urazowka.piekary.pl; 6 Department of Radiology, Inselspital, Bern University Hospital, University of Bern, Freiburgstrasse 10, 3010 Bern, Switzerland; andreas.christe@insel.ch

**Keywords:** anatomy, disproportional spine, disproportionate motion segment, disc herniation, lumbar spine, hypoplastic vertebral body

## Abstract

Background: The aim of this study was to determine whether the presence of disproportionate vertebral bodies is a risk factor for disc herniation (DH). Methods: Sixty-seven consecutive patients (m: 31 f: 36) who underwent lumbar discectomy for symptomatic DH at one level between L3 and S1 were retrospectively included. The last three motion segments (3 × 67 = 201) were assessed on sagittal MRI scans. A disproportionate motion segment was defined as the difference of more than 10% of the antero-posterior diameter of two adjacent endplates. Results: DH was present in 6/67 (9%), 26/67 (38.8%), and 35/67 (52.2%) patients at L3/4, L4/5, and L5/S1, respectively. A total of 14 of 67 patients demonstrated a disproportionate motion segment at the discectomy level (20.9%). A total of 23 of the 201 (11.4%) investigated motion segments met our criteria for a disproportionate motion segment. In our study population, when one of the 201 segments was disproportionate, the positive predictive value (PPV) for DH increased toward the lower segments: the PPV at the L5/S1 level was 83.0%. The odds ratio of disproportion for DH was the highest at the L5/S1 level, with 6.0 ± 0.82 (*p* = 0.017). Conclusions: The presence of a disproportionate motion segment in the lower spine may lead to a significant higher risk for DH in patients undergoing discectomy.

## 1. Introduction

Low back pain is one of the most common health issues in our society. Each year, 20–44% of the working population is affected, and the lifetime recurrence rate is as high as 85% [1,2,3]. There are many causes of low back pain, such as spondylolisthesis, lumbar spine stenosis, and disc herniation (DH). The incidence of DH is approximately 5–20 cases per 1000 adults per year. Most patients are between 30–50 years old, and men are more likely to be affected than are women (ratio 2:1) [4]. Most DH cases are located in the lower lumbar spine (95% at L4/5 and L5/S1). DH at higher levels is more commonly found in people older than 55 years [5]. The established risk factors for the occurrence of general low back pain include an older age, smoking, diabetes, a heavy body weight, and low levels of back and abdominal muscle strength. There is also a link between the occurrence of low back pain and psychological factors, such as anxiety, depression, and emotional instability. Regarding occupational factors, heavy work, lifting, bending, twisting, pulling, and pushing play a role in the development of low back pain [2,6].

The main requirement for the development of atraumatic DH is a degenerated disc. It is still not fully understood how the disc degenerates [7,8,9,10,11,12,13]. Anatomical studies have shown that the midsagittal diameter increases progressively from L1 to L5 [14,15,16,17,18,19]. Vertebral hypoplasia was defined as occurring when the AP diameter of a lower vertebral body was smaller than the adjacent upper vertebral diameter [20]. The phenomenon of hypoplasia of the lumbar vertebral body simulates spondylolisthesis [21] or osteoporotic fractures [22]. They measured the AP diameter of the middle of the vertebral body on sagittal MR images and reported the differences in the ratio (percentage) between two consecutive vertebral bodies. In our daily clinical practice, we observed that in patients with a symptomatic herniated disc, the vertebral diameters do not always increase progressively toward S1, as expected. Therefore, our hypothesis was that the presence of a smaller caudal vertebral body in a motion segment is a risk factor for the development of DH. The purpose of this study was to investigate the prevalence of disproportionate motion segments and to what extent this condition affects the risk for DH in patients undergoing discectomy. Furthermore, we aimed to determine normal segmental differences and find cutoff levels for disproportion.

## 2. Materials and Methods

### 2.1. Study Design

For our investigation, we chose a retrospective case study design. We searched our operation database for patients who underwent a discectomy from January 2014 to June 2018. The written consent of all patients was signed for the future use of their irreversibly anonymized images and data for research purposes. IRB was not in charge of retrospective studies containing irreversibly anonymized data at our institution.

### 2.2. Definition of DH

Localized displacement of disc material beyond the normal margins of the intervertebral disc space results in pain, weakness, or numbness in a myotomal or dermatomal pattern [23,24]. The presence of herniation or the bulging of a disk on MR images without clinical symptoms were not considered indicative of DH in our study.

### 2.3. Inclusion and Exclusion Criteria

The inclusion criteria for this study were patients (1) who underwent a discectomy for symptomatic DH after (2) failed conservative treatment (physiotherapy, steroid injection, and pain medications) and (3) demonstrated DH on MRI prior to the operation (<3 months) from L3 to S1 from January 2014 to June 2018 (*n* = 71). Some of the disproportionate segments were adjacent to the symptomatic level that was operated on; therefore, we measured the lowest three segments in all the included patients. Patients who had traumatic DH, spondylolisthesis, or other conditions leading to disc degeneration, such as instability, infection, tumor/metastasis, a previous related spine surgery, structural scoliosis, or congenital abnormalities of the spine, were excluded (*n* = 4). Age, sex, height, weight, and BMI were retrieved from the patient files. In addition, clinical data on smoking history and diabetes were collected since both are known risk factors for spine degeneration/DH [2,6].

### 2.4. Indications for Surgery

The patients in our outpatient clinic with low back pain with or without radiculopathy underwent an MRI scan of the lumbar spine when a herniated disc was suspected. The patients suffering from DH received conservative treatment for at least 3 months: physical therapy with a structured exercise program and a peri-radicular and/or epidural steroid injection (1 mL Kenacort (40 mg) and 1 mL Bupivacain (0.25%)) were prescribed. If the treatment was not successful and the patient still had clinically relevant impairments including motor deficiency (>M3 according to the Medical Research Council (MRC) muscle grading system [25]: 0, paralysis; 1, only a trace or flicker of muscle contractions is seen or felt; 2, muscle movement is possible in the absence of gravity; 3, muscle movement is possible in the presence of gravity; 4, muscle strength is reduced, but movement against resistance is possible; 5, normal muscle strength), the next step was to perform discectomy North American Spine Society: Clinical Guidelines for Diagnosis and Treatment of Lumbar Disc Herniation with Radiculopathy [24]). Patients with <M3 motor deficiency who underwent peracute operations were excluded due to a lack of chronic development.

### 2.5. Image Acquisition

The MR imaging scans were performed with a 1.5 T unit (MAGNETOM Aera, Siemens Healthineers, Erlangen, Germany) or a 3 T unit (Philips Ingenia, Philips Healthcare, Amsterdam, The Netherlands). The sequences included sagittal T1-weighted (TR/TE: 529/8) and T2-weighted (TR/TE: 3387/120) images with the 1.5 T unit and T1-weighted (TR/TE: 670/9.8) and T2-weighted (TR/TE: 6000/113) images with the 3 T MRI unit. For both sequences and scanners, a slice thickness of 3 mm was used.

### 2.6. Measurement

**Figure 1:**
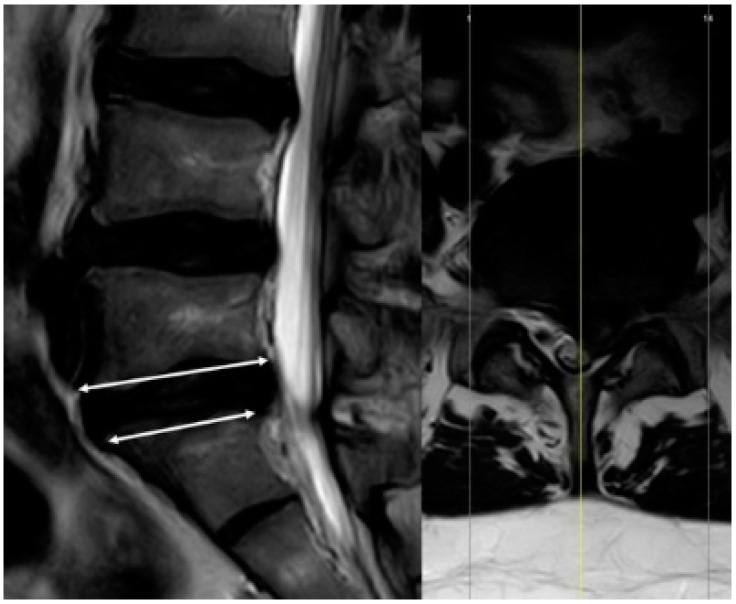
Location of measurement.

**Figure 2:**
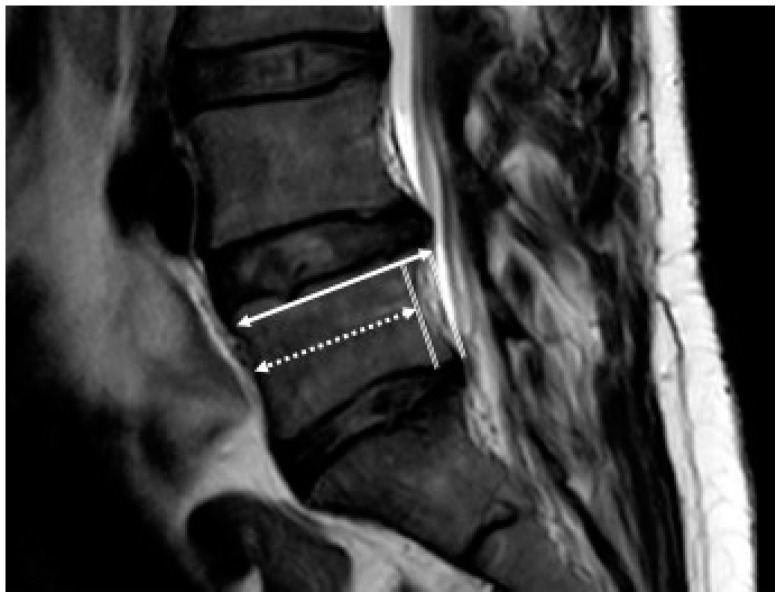
Difference in AP length in scalloping vertebrae.

**Figure 3:**
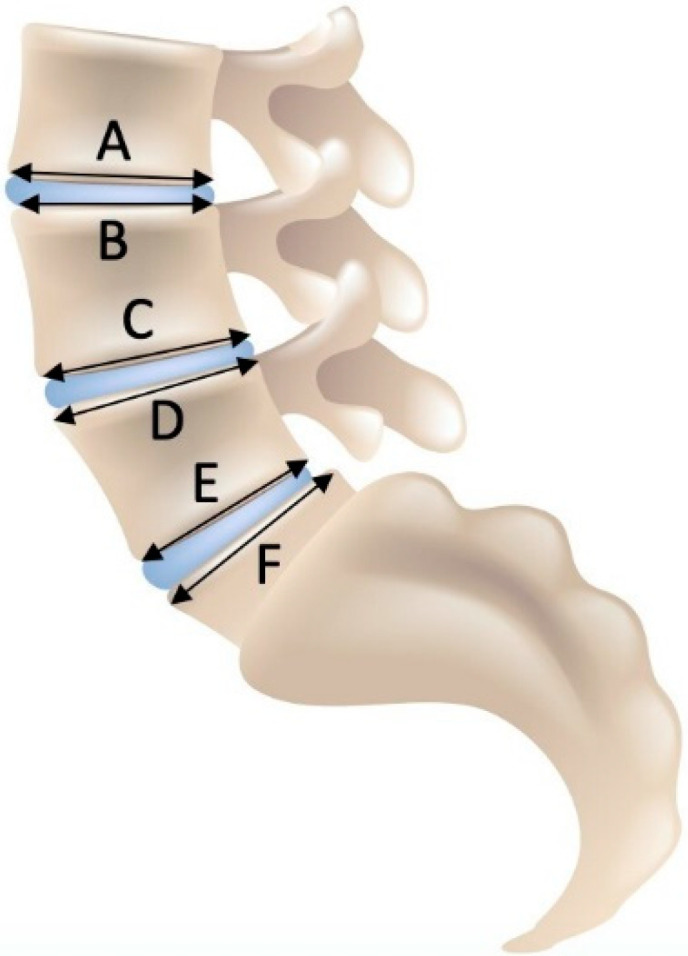
Schematic figure demonstrating the locations of the measurements. (**A**) inferior L3 endplate; (**B**) superior L4 endplate; (**C**) inferior L4 endplate; (**D**) superior L5 endplate; (**E**) inferior L5 endplate; (**F**) endplate S1.

**Figure 4:**
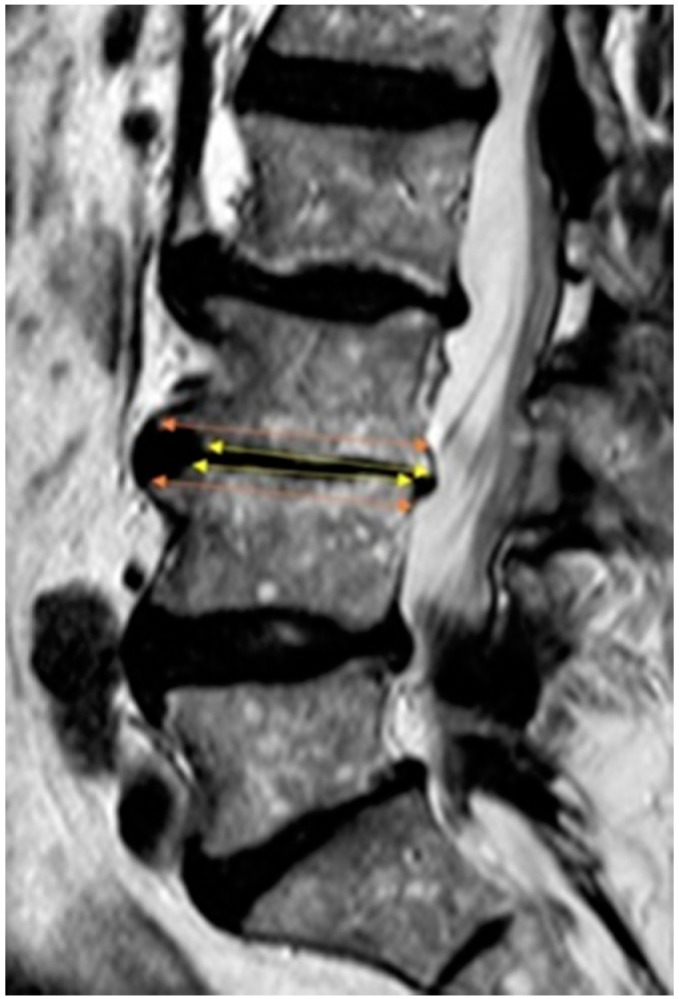
Only the effective bony area (yellow arrows) was measured compared to the maximum length (orange arrows).

Measurements were performed on the sagittal view on the T2-weighted images. The AP diameter of the cranial and caudal endplates of the spinal vertebrae at the midsagittal level from L3 to S1 was assessed (Figure 1). For each patient, we measured three motion segments for a total of 201 motion segments. Frank and Miller [21] and Wilms et al. [26] assessed the middle of the vertebra. However, scalloping vertebral bodies, shown in Figure 2, confounded the results; therefore, a modified measuring approach was used to compare the two adjacent endplates as shown in Figure 3; only the effective, weight-bearing, bony area was measured and is shown in Figure 4. The diameter was measured from the external borders of the vertebral body rims, thus excluding any osteophytes (Figure 4) so that the original anatomy could be assessed. All measurements of the 201 segments were performed by one orthopedic fellow in spine surgery (R.B.) with four years of experience performing surgery. For interobserver agreement (reproducibility), a second consultant (T.P.) with five years of experience in spine surgery examined 30 randomized disc segments. To assess the level of intraobserver variability, the same 30 segments were remeasured by both raters after an interval of one month. All measurements were performed with a picture archiving and communication system (Phoenix GmbH, Freiburg im Breisgau, Germany) using an electronic caliber.

### 2.7. Theory/Calculations

The AP diameters of the caudal endplate of the upper vertebral body and the cranial endplate of the lower vertebral body were used to determine whether disproportion existed (the length of the caudal endplate minus the length of the cranial endplate from the consecutive vertebral body). In 2012, Niggemann et al. [27] reported the cutoff value for disproportion to be 3 mm because this value corresponds to the level of variance in MRI measurements of vertebral bodies. For a more accurate analysis of disproportion, the diameter difference was normalized to the absolute vertebral endplate diameter. Therefore, we defined the endplate difference cutoff value to be 10% for a disproportionate motion segment and calculated the degree of disproportion (DP) as follows using Equation (1):

DP = (A − B)/A(1)

The degree of DP was calculated as the difference in diameter between the lower endplate of the upper vertebral body (A) and the cranial endplate of the lower vertebral body (B) normalized to (A), as shown in Figure 3.

The kappa (κ) values for interobserver and intraobserver agreement for both raters were calculated with respect to DP. The following Kappa classification of agreement was used: κ < 0 = poor agreement, 0–0.20 = slight agreement, 0.21–0.40 = fair agreement, 0.41–0.60 = moderate agreement, 0.61–0.80 = substantial agreement, and 0.81–1.00 = (almost) perfect agreement: There was almost perfect intraobserver agreement (κ= 0.938). Reproducibility between the two raters was high, with a substantial interobserver agreement value of κ = 0.739.

### 2.8. Statistical Analysis

(1) Anatomical parameters were assessed per segment. The significance of the differences between adjacent endplate distances was assessed with the paired Wilcoxon test.

(2) The prevalence of DH was calculated for disproportionate and regular motion segments. The positive predictive value (PPV) and odds ratios of disproportion for the detection of DH were calculated. The chi-square test was performed to compare the amount of disproportion and DH at all discectomy levels and all pooled segments from L3 to S1. A sub-analysis of cases with disc sequestration (DS) was performed to investigate the association between disproportion and sequestration (Fisher exact test). MR-images were reviewed by R.B. and A.C.

(3) Odds ratios and the corresponding 95% confidence intervals (CI) were calculated. Logistic regression was used to test selected variables as shown in Equation (2):

(DH) = b0 + c1∗A + c2∗W + c3∗H + c4∗B + c5∗M + c6∗D + c7∗S + c8∗DP(2)

In the equation above: (A) age, (W) weight, (H) height, (B) BMI, (M) sex, (Y/N) diabetes, (Y/N) smoking, and (DP) disproportion were analyzed to predict DH. In equation (2), b0 is a constant, and c1 to c8 are the coefficients for each variable. In the logistic regression model, the dependent variable was binary (disc herniation yes/no). The independent variable inputs are categorical (male/female sex, diabetes y/n, smoking y/n, and disproportion y/n) or continuous numbers (age, weight, height, BMI) using MedCalc (version 7.6.0.0, Ostend, Belgium).

(4) The correlation coefficient r between DP and DH and the receiver operating characteristic (ROC) curve for the best cutoff value for DP for predicting DH were determined, and the area under the curve (AUC) was calculated.

MedCalc (version 7.6.0.0, Ostend, Belgium) was used for the statistical analyses. *p* < 0.05 indicated statistical significance.

## 3. Results

### 3.1. Study Population

Sixty-seven consecutive patients met our inclusion criteria (M:F = 31:36). The patient characteristics are shown in Table 1.

### 3.2. Anatomical Results

**Figure 5:**
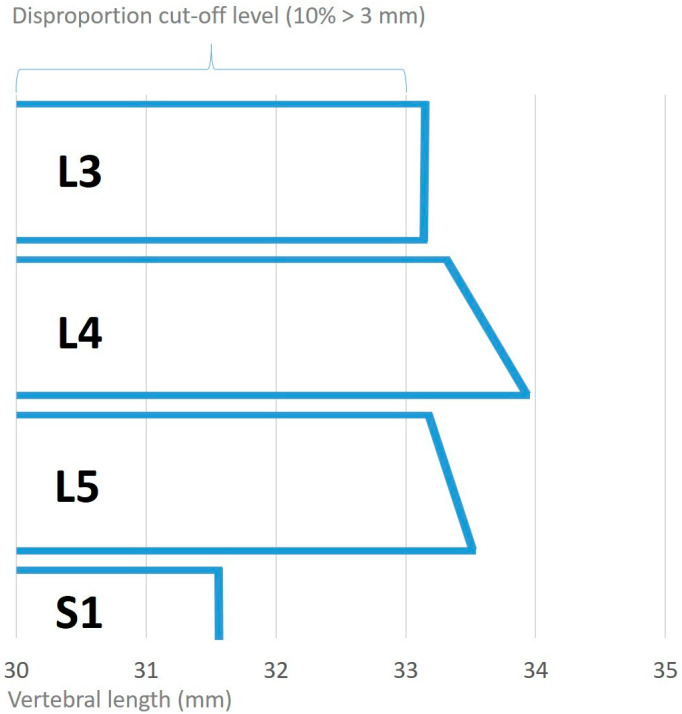
Descriptive anteroposterior (AP) measurements of the vertebral endplates in mm in our study population. S1 is smaller than L5, and L5 is smaller than L4. The 10% cutoff definition for disproportion is also displayed.

In our study population, the mean length of the upper vertebral endplate was the same length or shorter than that of the lower endplate of the same vertebral body. The endplates of the adjacent vertebrae increased at segments L3 to L4 and decreased significantly from L4 to L5 and from L5 to S1 (Table 2). The differences in length were clearly below the 10% cutoff value for disproportion Figure 5.

### 3.3. Segmental Distribution of DH in the Study Population (67 Discectomies)

DH was present in 6/67 (9%), 26/67 (38.8%), and 35/67 (52.2%) patients at L3/4, L4/5, and L5/S1, respectively. A total of 14 out of 67 patients demonstrated a disproportionate motion segment at the discectomy level (20.9%).

### 3.4. Segmental Distribution of Disproportionate Motion Segments

A total of 23 of the 201 (11.4%) investigated motion segments (three motion segments per patient; 3 × 67 = 201) met our criteria for a disproportionate motion segment. In our study population, the distribution of disproportionate motion segments increased from the cranial to caudal direction: 3/67 (4.5%), 8/67 (12%), and 12/67 (18%) disproportionate segments were present at L3/4, L4/5, and L5/S1, respectively.

### 3.5. Disproportion as a Risk Factor for DH

There were significantly more disproportionate segments in the presence of DH (14/67) than in the absence of DH (9/134) (*p*-value = 0.003). When disproportion was present in one of the pooled 201 segments, the PPV for DH increased toward the lower segments: the PPV at the L5/S1 level was 83.0%. The ratio of the odds for DH in the presence of disproportion and the odds for DH in the absence of disproportion was the highest at the L5/S1 level, with the value being 6.0 ± 0.82 (*p* = 0.017). The statistical results stratified by level are shown in Table 3.

### 3.6. Logistic Regression Correlation and ROC Curve between DP and DH

Only one variable, disproportion, tested positive for predicting DH, with an odds ratio of 4.11 (CI: 1.60 to 10.54, *p* = 0.003). The other variables are listed in Table 4. There was a significant correlation between DP and DH, with a correlation coefficient r of 0.21 (CI: 0.07 to 0.34; *p*-value = 0.0028). The best accuracy for predicting DH was reached using a cutoff value for disproportion of >2.7%, leading to a sensitivity and specificity of 65.7% and 67.9% for DH, respectively, and an AUC of 0.639.

### 3.7. Disc Sequestration (DS)

Thirty patients out of the 67 DH presented with a disc sequestration (DS = 44.8%). From the 30 DS, 4 demonstrated a disproportion (13.3%), while 26 had no disproportion (86.7%). The ratio of disproportion was not significantly different in the 37 DH without DS (*p* = 0.231): 10 patients showed disproportion (27.0%), and 27 did not have disproportion (73.0%).

## 4. Discussion

Disproportionate vertebral body configurations are frequently seen on MR images. Few studies have investigated the prevalence of disproportionate vertebral bodies on lumbosacral MR images in symptomatic DH patients. The clinical relevance of this radiological phenomenon has not been addressed in the literature thus far. We showed that the incidence of such a difference in length is particularly frequent in the distal part of the lumbar spine. Hypoplastic vertebral bodies were previously diagnosed by visual assessment of the alignment on conventional X-rays [21] or by measuring the vertebral bodies on mid-vertebral level on MRI [26]. In the presence of scalloping vertebral bodies, this method can underestimate the true level of bony support for the intervertebral disc (Figure 2). Therefore, a modified measuring approach was used to compare the two adjacent endplates (Figure 3); only the effective, weight-bearing, bony area was measured (Figure 4). A cutoff value of endplate difference of 3 mm was reported for disproportion because this value corresponded to the level of variance in MRI measurements of vertebral bodies [27]. For a more accurate analysis of disproportion, the diameter difference was normalized to the absolute vertebral endplate diameter (~30 mm). Therefore, we defined the cutoff value of endplate difference to be 10% for a disproportionate motion segments.

Different studies have suggested that the AP diameters of vertebrae increase from the cervical to lumbar direction [16,17,18]. In 2008, Masharawi et al. [28] stated that the superior vertebral length continuously increases from T1 to L5 and that the inferior vertebral length increases from T1 to L4 and then slightly decreases at L5. The superior vertebral length is usually larger than the inferior vertebral length of the adjacent superior vertebra (except at T2–T3 and T11–T12) [28]. The results of our study do not confirm this finding for the lowest two lumbar segments, as we found that the endplate distance between the adjacent endplates decreased in the inferior direction. In our study population, L5 was smaller than L4, and S1 was smaller than L5 (*p* < 0.0001). However, mean measurement differences of up to 2 mm (<6%) were not considered as disproportion according to our definition (>10%).

The ROC analysis revealed a cutoff level of >2.7% for disproportion (equaling~0.8 mm) for the prediction of DH. This value lies below our threshold of 10%, based on the measurement variance in previous studies [27]. This finding demonstrates that disproportion is relevant (especially with an AUC of 0.639), but this cutoff level is probably not recommended and does not correspond to a real disproportionate segment.

Vertebral disproportion is an important finding and was found to be a risk factor for DH. Compared to all other known risk factors, vertebral disproportion was the only variable that could predict DH. However, disproportion was not associated with disc sequestration. Not only for surgeons but also for radiologists, disproportion is an important imaging finding, and it may be detected by MRI as well as conventional and CT imaging. Additional comparative studies need to be performed.

Disproportion has long been misinterpreted as retrolisthesis but is in fact indicative of a hypoplastic vertebral body. From a biomechanics point of view, early degeneration occurs when the weight cannot be distributed evenly across the surfaces between vertebrae, and degeneration occurs faster in cases of scoliosis. This sign of disproportion can indicate that surgical treatment cannot be avoided despite the patient undergoing the best conservative treatment. This finding may help patients suffering from DH decide whether to undergo an operation and may be included as an indication for surgery in the future after additional evaluations.

For simplicity reasons, an easier measurement formula for disproportion can be used: B/A identifies exactly the same disproportionate motion segments as the formula we used when a cutoff level of 90% is used.

## 5. Limitations

Measurements made on CT images may be even more accurate than those made on MR images due to a higher bone soft tissue contrast, and this topic needs to be studied further.

Not all potential risk factors could be examined, and this study did not include patients undergoing conservative therapy, which may pose a bias.

The statistical power of an analysis including 201 individual patients would be higher than that of the analysis of three segments in 67 patients regarding the prevalence of DP. One could argue that a patient with a DP at one segment is prone to having other disproportionate segments or anatomical anomalies.

## 6. Conclusions

Disproportionate motion segments in the lower spine may lead to a significant higher risk for DH in patients undergoing discectomy.

## Figures and Tables

**Table 1:** Study population.

Population		*n*	%
**All Patients**		67	100
Sex	Female	36	50.7
	Male	31	49.3
Age (years)	median (range)	49 (18–87)	
Body Height (cm)	median (range)	172 (153–191)	
Body Weight (kg)	median (range)	74 (50–155)	
Body Mass Index (BMI)	median (range)	25 (18.8–49.5)	
Smoker		24	35.8
Diabetes		2	3.0

**Table 2:** Summary statistics of the endplate measurements.

Vertebral Endplate Level	*n*	Minimum (mm)	Maximum (mm)	Mean (mm)	Median (mm)	SD	*p*-Value
upper L3	67	25.8	46.6	33.2	33.3	3.3	0.9875
lower L3	67	25.1	42.9	33.2	33.2	3.4	0.2279
upper L4	67	27.2	41.8	33.3	33.3	3.1	0.0034
lower L4	67	27.1	44.7	34.0	34.4	3.4	0.0146
upper L5	67	27.1	41.1	33.2	33.1	3.3	0.2365
lower L5	67	26.4	41.2	33.5	33.6	3.5	<0.0001
upper S1	67	24.6	39.9	31.6	31.1	3.5	n/a

Abbreviations: SD, standard deviation; n/a, not available; *p*-value, compared to the next lower endplate.

**Table 3:** The status of disproportion and DH at each disc level.

L3/4	DH+	DH−	PPV	OR	±SE	*p*-Value
Disproportion +	0	3	0.00	n/a	n/a	1.000
Disproportion −	6	58				
**L4/5**	**DH+**	**DH−**	**PPV**	**OR**	**±SE**	***p*** **-Value**
Disproportion +	4	4	0.50	1.68	0.76	0.489
Disproportion −	22	37				
**L5/S1**	**DH+**	**DH−**	**PPV**	**OR**	**±SE**	***p*** **-Value**
Disproportion +	10	2	0.83	6.00	0.82	0.017
Disproportion −	25	30				
**L3-S1 (pooled)**	**DH+**	**DH−**	**PPV**	**OR**	**±SE**	***p*** **-Value**
Disproportion +	14	9	0.61	3.67	0.46	0.003
Disproportion −	53	125				

Abbreviations: DH, disc herniation; PPV, positive predictive value; OR, odds ratio; SE, standard error; n/a, not available.

**Table 4:** Logistic regression of disc herniation with disproportion (DP) and potential confounding variables.

Variable	Coefficient c1–8	Std. Error	Odds Ratio	95% CI	*p*
Age (A)	0.01	0.01	1.01	0.99 to 1.03	0.520
Weight (W)	0.04	0.10	1.04	0.85 to 1.28	0.721
Height (H)	−0.03	0.10	0.97	0.81 to 1.18	0.787
BMI (B)	−0.11	0.31	0.90	0.49 to 1.66	0.728
Male sex (M)	−0.07	0.45	0.93	0.39 to 2.25	0.874
Diabetes (D)	−0.61	1.41	0.54	0.03 to 8.65	0.665
Smoking (S)	−0.03	0.36	0.97	0.48 to 1.94	0.925
Disproportion (DP)	1.41	0.48	4.11	1.60 to 10.54	0.003
Constant b_0_	3.28	16.78			0.845

CI, confidence interval; *p*, *p*-value.

## Data Availability

Data are available upon special request.

## References

[1] Maniadakis N., Gray A. (2000). The Economic Burden of Back Pain in the UK. Pain.

[2] Woolf A.D., Pfleger B. (2003). Burden of Major Musculoskeletal Conditions. Bull. World Health Organ..

[3] Hoy D., Bain C., Williams G., March L., Brooks P., Blyth F., Woolf A., Vos T., Buchbinder R. (2012). A Systematic Review of The Global Prevalence of Low Back Pain. Arthritis Rheum..

[4] Leven D., Passias P.G., Errico T.J., Lafage V., Bianco K., Lee A., Lurie J.D., Tosteson T.D., Zhao W., Spratt K.F. (2015). Risk Factors for Reoperation in Patients Treated Surgically for Intervertebral Disc Herniation: A Subanalysis of Eight-Year SPORT Data. J. Bone Jt. Surg. Am..

[5] Jordon J., Konstantinou K., O’Dowd J. (2009). Herniated Lumbar Disc. BMJ Clin. Evid..

[6] Kushchayev S.V., Glushko T., Jarraya M., Schuleri K.H., Preul M.C., Brooks M.L., Teytelboym O.M. (2018). ABCs of The Degenerative Spine. Insights Imaging.

[7] Modic M.T., Ross J.S. (2007). Lumbar Degenerative Disk Disease. Radiology.

[8] Vergroesen P.P., Kingma I., Emanuel K.S., Hoogendoorn R.J., Welting T.J., van Royen B.J., van Dieën J.H., Smit T.H. (2015). Mechanics and Biology in Intervertebral Disc Degeneration: A Vicious Circle. Osteoarthr. Cartil..

[9] Stefanakis M., Luo J., Pollintine P., Dolan P., Adams M.A. (2014). ISSLS Prize Winner: Mechanical Influences in Progressive Intervertebral Disc Degeneration. Spine.

[10] Russo F., Ambrosio L., Ngo K., Vadalà G., Denaro V., Fan Y., Sowa G., Kang J.D., Vo N. (2019). The Role of Type I Diabetes in Intervertebral Disc Degeneration. Spine.

[11] Urban J.P., Smith S., Fairbank J.C. (2004). Nutrition of The Intervertebral Disc. Spine.

[12] Urban J.P., Roberts S. (2003). Degeneration of The Intervertebral Disc. Arthritis Res. Ther..

[13] Hadjipavlou A.G., Tzermiadianos M.N., Bogduk N., Zindrick M.R. (2008). The Pathophysiology of Disc Degeneration: A Critical Review. J. Bone Jt. Surg. Br..

[14] Kanna R.M., Shetty A.P., Rajasekaran S. (2014). Patterns of Lumbar Disc Degeneration Are Different in Degenerative Disc Disease and Disc Prolapse Magnetic Resonance Imaging Analysis of 224 Patients. Spine J..

[15] Steffens D., Hancock M.J., Maher C.G., Williams C., Jensen T.S., Latimer J. (2014). Does Magnetic Resonance Imaging Predict Future Low Back Pain? A Systematic Review. Eur. J. Pain.

[16] Berry J.L., Moran J.M., Berg W.S., Steffee A.D. (1987). A Morphometric Study of Human Lumbar and Selected Thoracic Vertebrae. Spine.

[17] Wang T.M., Shih C. (1992). Morphometric Variations of The Lumbar Vertebrae Between Chinese and Indian Adults. Cells Tissues Organs.

[18] Davies K.M., Recker R.R., Heaney R.P. (1989). Normal Vertebral Dimensions and Normal Variation in Serial Measurements of Vertebrae. J. Bone Min. Res..

[19] Williams P.L., Bannister L.H., Berry M.M., Collins P., Dyson M., Dussek J.E., Ferguson M.W. (1995). Gray’s Anatomy: The Anatomical Basis of Medicine and Surgery.

[20] Kim S.K., Lee S.R., Moon W.J., Park D.W., Hahm C.K. (2000). Regional Disc Change in Segmental Hypoplasia of The Lumbosacral Vertebral Bodies: MR Findings. J. Korean Radiol. Soc..

[21] Frank D.F., Miller J.E. (1979). Hypoplasia of The Lumbar Vertebral Body Simulating Spondylolisthesis. Radiology.

[22] Oura P., Nurkkala M., Auvinen J., Niinimäki J., Karppinen J., Junno J.A. (2019). The Association of Body Size, Shape and Composition with Vertebral Size in Midlife–The Northern Finland Birth Cohort 1966 Study. Sci. Rep..

[23] Abdel-Salam A., Eyres K.S., Cleary J. (1992). Management of The Herniated Lumbar Disc: The Outcome After Chemonucleolysis, Surgical Disc Excision and Conservative Treatments. Eur. Spine J..

[24] www.spine.org. https://www.spine.org/Portals/0/Assets/Downloads/ResearchClinicalCare/Guidelines/LumbarDiscHerniation.pdf.

[25] Vanhoutte E.K., Faber C.G., Van Nes S.I., Jacobs B.C., Van Doorn P.A., Van Koningsveld R., Cornblath D.R., Van Der Kooi A.J., Cats E.A., Van Den Berg L.H. (2012). Modifying the Medical Research Council Grading System through Rasch Analyses. Brain.

[26] Wilms G., Maldague B., Parizel P., Meylaerts L., Vanneste D., Peluso J. (2009). Hypoplasia of L5 and Wedging and Pseudospondylolisthesis in Patients with Spondylolysis: Study With MR Imaging. AJNR Am. J. Neuroradiol..

[27] Niggemann P., Kuchta J., Grosskurth D., Beyer H.K., Hoeffer J., Delank K.S. (2012). Spondylolysis and Isthmic Spondylolisthesis: Impact of Vertebral Hypoplasia on The Use of The Meyerding Classification. Br. J. Radiol..

[28] Masharawi Y., Salame K., Mirovsky Y., Peleg S., Dar G., Steinberg N., Hershkovitz I. (2008). Vertebral Body Shape Variation in The Thoracic and Lumbar Spine: Characterization of Its Asymmetry and Wedging. Clin. Anat..

